# Mutation of the SUMOylation site of Aurora-B disrupts spindle formation and chromosome alignment in oocytes

**DOI:** 10.1038/s41420-024-02217-7

**Published:** 2024-10-22

**Authors:** Shan-Shan Chen, Li Li, Bo Yao, Jia-Lun Guo, Ping-Shuang Lu, Hao-Lin Zhang, Kun-Huan Zhang, Yuan-Jing Zou, Nan-Jian Luo, Shao-Chen Sun, Lin-Lin Hu, Yan-Ping Ren

**Affiliations:** 1 Department of Histology and Embryology, School of Preclinical Medicine, Zunyi Medical University, Zunyi, Guizhou 563000 China; 2 College of Animal Science and Technology, Nanjing Agricultural University, Nanjing, Jiangsu 210095 China; 3 Department of Reproduction, Suzhou TCM Hospital Affiliated to Nanjing University of Chinese Medicine, Suzhou, Jiangsu 215000 China; 4 Key Laboratory of Research on Clinical Molecular Diagnosis for High Incidence Diseases in Western Guangxi, Affiliated Hospital of Youjiang Medical University for Nationalities, Baise, Guangxi 533000 China

**Keywords:** Meiosis, Post-translational modifications

## Abstract

Aurora-B is a kinase that regulates spindle assembly and kinetochore-microtubule (KT-MT) attachment during mitosis and meiosis. SUMOylation is involved in the oocyte meiosis regulation through promoting spindle assembly and chromosome segregation, but its substrates to support this function is still unknown. It is reported that Aurora-B is SUMOylated in somatic cells, and SUMOylated Aurora-B contributes the process of mitosis. However, whether Aurora-B is SUMOylated in oocytes and how SUMOylation of Aurora-B impacts its function in oocyte meiosis remain poorly understood. In this study, we report that Aurora-B is modified by SUMOylation in mouse oocytes. The results show that Aurora-B colocalized and interacted with SUMO-2/3 in mouse oocytes, confirming that Aurora-B is modified by SUMO-2/3 in this system. Compared with that in young mice, the protein expression of SUMO-2/3 decreased in the oocytes of aged mice, indicating that SUMOylation might be related to mouse aging. Overexpression of Aurora-B SUMOylation site mutants, Aurora-B^K207R^ and Aurora-B^K292R^, inhibited Aurora-B recruitment and first polar body extrusion, disrupting localization of gamma tubulin, spindle formation and chromosome alignment in oocytes. The results show that it was related to decreased recruitment of p-HDAC6 which induces the high stability of whole spindle microtubules including the microtubules of both correct and wrong KT-MT attachments though increased acetylation of microtubules. Therefore, our results corroborate the notion that Aurora-B activity is regulated by SUMO-2/3 in oocytes, and that SUMOylated Aurora B plays an important role in spindle formation and chromosome alignment.

## Introduction

It is estimated that 20% of human oocytes were aneuploid, and this proportion increased exponentially from 30 to 35 years, averaging 80% by 42 years [1]. Unlike spermatocytes, mammalian oocytes undergo two rounds of developmental arrest during maturation: the first meiotic division produces a fertilizable egg, while the second meiotic division occurs upon fertilization [2, 3]. Unfaithful chromosome segregation of oocytes caused by developmental arrest is one of the major causes of female infertility [4, 5]. Microtubules are cytoskeletal components that play significant roles in directed cell migration, vesicle and organelle transport and mitosis. Among numerous microtubule-regulating proteins, gamma tubulin (γ-tubulin) is a key component involved in the nucleation and organization of microtubules [6]. Tubulin acetylation is a posttranslational modification that is crucial for regulating microtubule architecture and maintaining microtubule integrity [6, 7]. Histone deacetylase 6 (HDAC6) is a unique cytoplasmic deacetylase that regulates the deacetylation of α-tubulin, which regulates spindle formation or oocyte maturation [8, 9]. These meiotic maturation processes in oocytes are precisely regulated by various protein kinase networks and posttranslational modifications [10–12].

SUMOylation is a reversible posttranslational modification that is involved in regulating various biological processes, such as cell cycle progression, DNA maintenance and repair and nucleocytoplasmic transport [13, 14]. In oocytes from *C. elegans*, SUMOylation regulates the assembly of ring complex, CPC assembly and the localization of AIR-2/Aurora-B kinase during meiosis [15]. Deficiency of the SUMO E2-conjugating enzyme (UBC9) and SUMO-specific protease 7 (SENP7) causes significant chromosome condensation and segregation abnormalities and decreases the first polar body (PB1) extrusion in mouse oocytes [16–19]. The overexpression of SENP2, a SUMO-specific isopeptidase, to change SUMO-modified proteins caused defects in MII spindle formation [18]. Furthermore, the depletion of SUMO-1 disrupted kinetochore-microtubule attachment in metaphase I of oocytes [19]. The above studies indicated that SUMOylation is necessary for meiosis in oocytes, but its substrates to support this function is still unclear.

Aurora-B is a member of the chromosomal passenger complex (CPC), which is composed of Aurora-B protein kinase, the inner centromere protein (INCENP), and the targeting subunits Survivin and Borealin [20]. Aurora-B is involved in correcting kinetochore-microtubule (KT-MT) attachment errors, chromosome alignment and the spindle assembly checkpoint (SAC) during mitosis [21, 22]. In oocyte meiosis, the treatment with the Aurora-B/C inhibitor caused chromosome misalignment and decreased PB1 extrusion [23, 24]. Aurora-B cKO increased the number of aneuploid metaphase II-arrested eggs and exhibited an age-related reduction in SAC integrity [25, 26]. The above studies indicate that Aurora-B is necessary for correcting chromosomal segregation in oocyte meiosis with an oocyte-age related manner. Moreover, Aurora-B is recruited and destroys the error KT-MT attachment in oocyte meiosis [27–29], suggesting the function in correcting kinetochore-microtubule (KT-MT) attachment errors. These results show there are similar functions of Aurora-B in both mitosis and meiosis [25, 26]. However, the activity of Aurora-B maintains even on correct KT-MT attachment in pre-MI oocytes and sustained still at late-MI stage [27–29], while Aurora-B is inactived at that situation in mitosis [30, 31]. It suggests that the mechanism that Aurora-B regulates the progress of cell division differs in some detail between mitosis and meiosis, which might explain the happen of oocyte aneuploidy [32].

Previous research showed that the SUMO modification of some key factor, such as CENP-E, is essential for mammalian cell mitosis [33]. Aurora-B is SUMOylated during mitosis [34]. Furthermore, it has been proved that Lys-207 site of Aurora-B is a major modification site in mitosis and SUMOylated Aurora-B induces the recruitment of itself to centromeres in prometaphase and metaphase of mitosis to promote cell proliferation [33–36]. However, whether Aurora-B can be SUMOylated, and the potential molecular mechanism involved in oocyte meiosis are unclear. In this study, we focused primarily on whether Aurora-B could be SUMOylated in mouse oocytes, as well as the molecular mechanism of SUMOylation of Aurora-B during oocyte meiosis.

## Results

### Aurora-B is SUMOylated by SUMO-2/3 in mouse oocytes

**Fig. 1:**
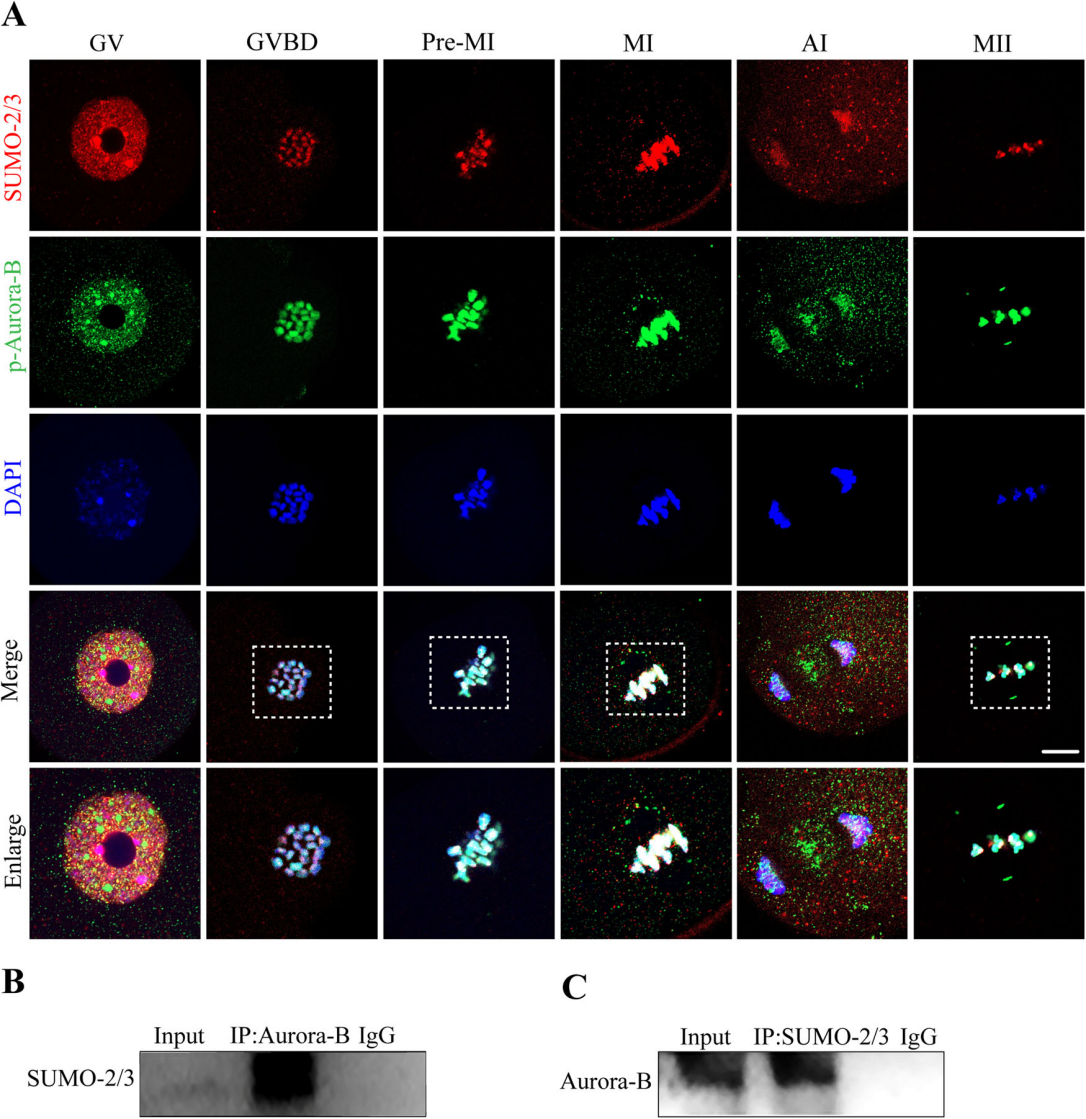
Aurora-B is SUMOylated by SUMO-2/3 in mouse oocytes. **A** Subcellular localization of Aurora-B and SUMO-2/3 in mouse oocytes at different phases. Mouse oocytes were matured and harvested in vitro at the following stages: GV (0 h), GVBD (4 h), pre-MI (6 h), MI (8 h), AI (10 h), and MII (16 h). Rabbit anti-p-Aurora-B (green), rat anti-SUMO-2/3 (red), and DAPI-labeled DNA (blue) were used. Scale bar: 5 μm. **B** Co-immunoprecipitation was performed with anti-Aurora-B and detected by WB using an anti-SUMO-2/3 antibody. **C** Co-immunoprecipitation was performed with anti-SUMO-2/3 and detected by WB using anti-Aurora-B.

The subcellular localization of Aurora-B and SUMO-2/3 in mouse oocytes at the germinal vesicle (GV), germinal vesicle breakdown (GVBD), premetaphase I (Pre-MI), metaphase I (MI), anaphase I (AI) and metaphase II (MII) stages was determined to evaluate the stages of potential Aurora-B SUMOylation. Aurora-B was localized to the nucleus of oocytes in GV oocytes. SUMO-2/3 was also mainly distributed in the nucleus. After GVBD, Aurora-B and SUMO-2/3 aggregated on chromatin regions. As oocytes enter the pre-MI and MI stages, Aurora-B colocalizes with SUMO-2/3 on chromosomes. In AI oocytes, Aurora-B was enriched in the spindle midzone and chromosomes. However, SUMO-2/3 was only localized on chromosomes. In MII oocytes, Aurora-B and SUMO-2/3 were localized to the chromosome arm region (Fig. 1A). The colocalization of Aurora-B and SUMO-2/3 changed cyclically, suggesting the possibility of interactions between pre-MI, MI and MII oocytes during meiosis.

To confirm this hypothesis, we next examined the protein interaction relationship between Aurora-B and SUMO-2/3 in pre-MI oocytes by immunoprecipitation. The results showed that SUMO-2/3 was immunoprecipitated by anti-Aurora-B (Fig. 1B and Fig. S3), and Aurora-B was detected in the immunoprecipitated sample of anti-SUMO-2/3 (Fig. 1C and Fig. S3). These findings showed that Aurora-B interacts with SUMO-2/3 and that Aurora-B colocalizes with SUMO-2/3, suggesting Aurora-B is SUMOylated by SUMO-2/3 in mouse oocytes.

### Age-related SUMO-2/3 expression in mouse oocytes

**Fig. 2:**
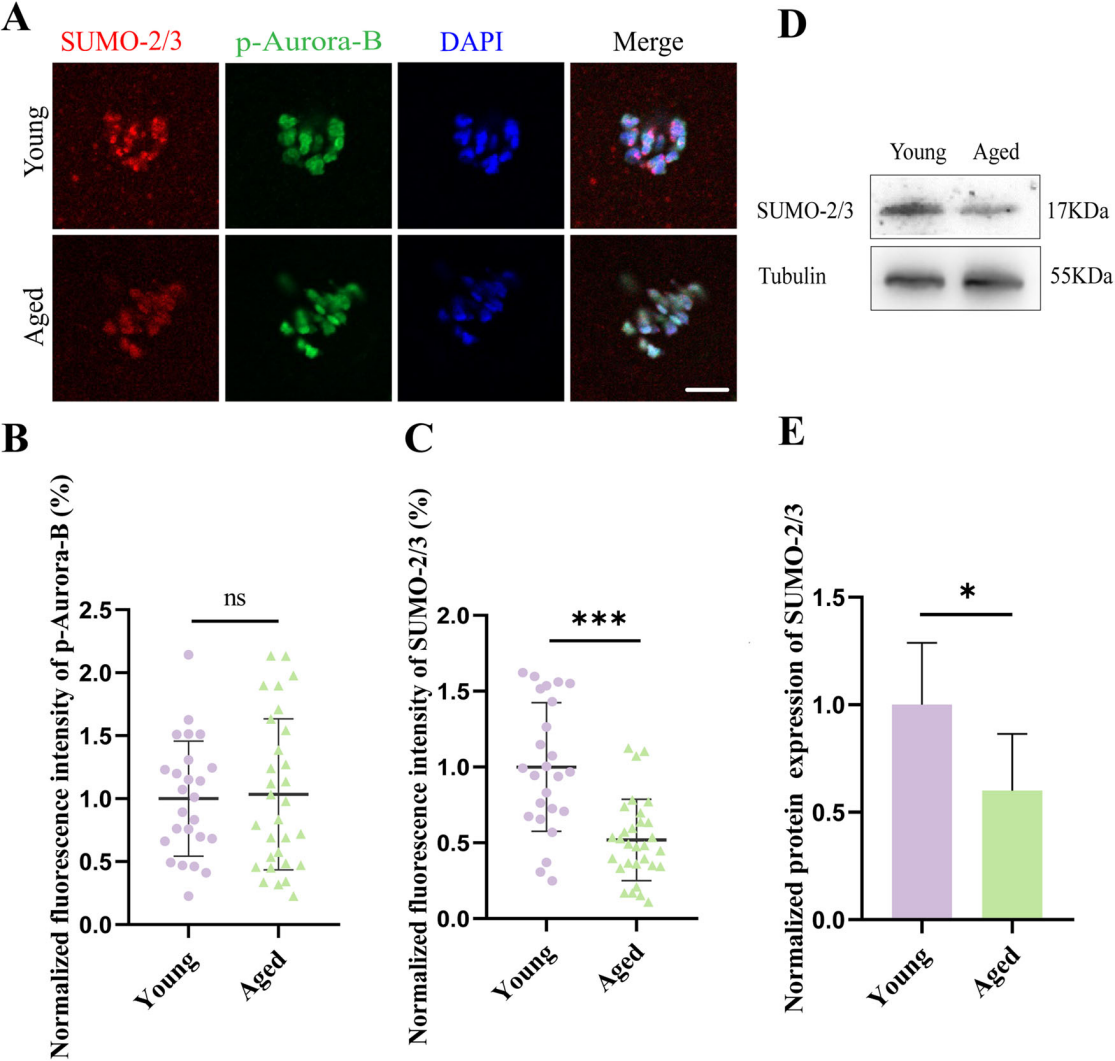
Age-related SUMO-2/3 expression in mouse oocytes. **A** Representative images of p-Aurora-B and SUMO-2/3 in pre-MI oocytes from young and old groups. Young: 6 weeks, Aged: 12 months. p-Aurora-B (green), SUMO-2/3 (red), and DAPI-labeled DNA (blue) were used. Scale bars: 5 μm. **B** The fluorescence intensity of p-Aurora-B in pre-MI oocytes from young and old mice. ns *P* > 0.05. **C** The fluorescence intensity of SUMO-2/3 in pre-MI oocytes from young and aged mice. ***P < 0.001. **D** Western blot analysis of SUMO-2/3 in pre-MI mouse oocytes in the young and old groups. **E** Statistical analysis of the protein expression of SUMO-2/3. *P < 0.05.

We then studied whether Aurora-B and SUMO-2/3 may be impacted by aging, and their fluorescence intensities were compared between the young and old groups. The statistical results showed that compared with that in the young group, the average fluorescence intensity of Aurora-B in the aged group did not differ (young: 1 ± 0.4569, n = 25 vs aged: 1.034 ± 0.5996, n = 30, *P* > 0.05) (Fig. 2A, B). However, the average fluorescence intensity of SUMO-2/3 decreased significantly in the aged group (young: 1 ± 0.4234, n = 25 vs aged: 0.5195 ± 0.2684, n = 30, *P* < 0.001) (Fig. 2A, C). Compared with that in the young group, SUMO-2/3 protein expression decreased in the oocytes of the aged group (young: 1 ± 0.2887, n = 3 vs aged: 0.6005 ± 0.2640, n = 3, *P* < 0.05) (Fig. 2D, E and Fig. S3). These data indicated that, although SUMOylation of Aurora B might be affected by aging, the effects on changes in Aurora-B activity were not found.

### SUMOylation is essential for Aurora-B recruitment to oocytes

**Fig. 3:**
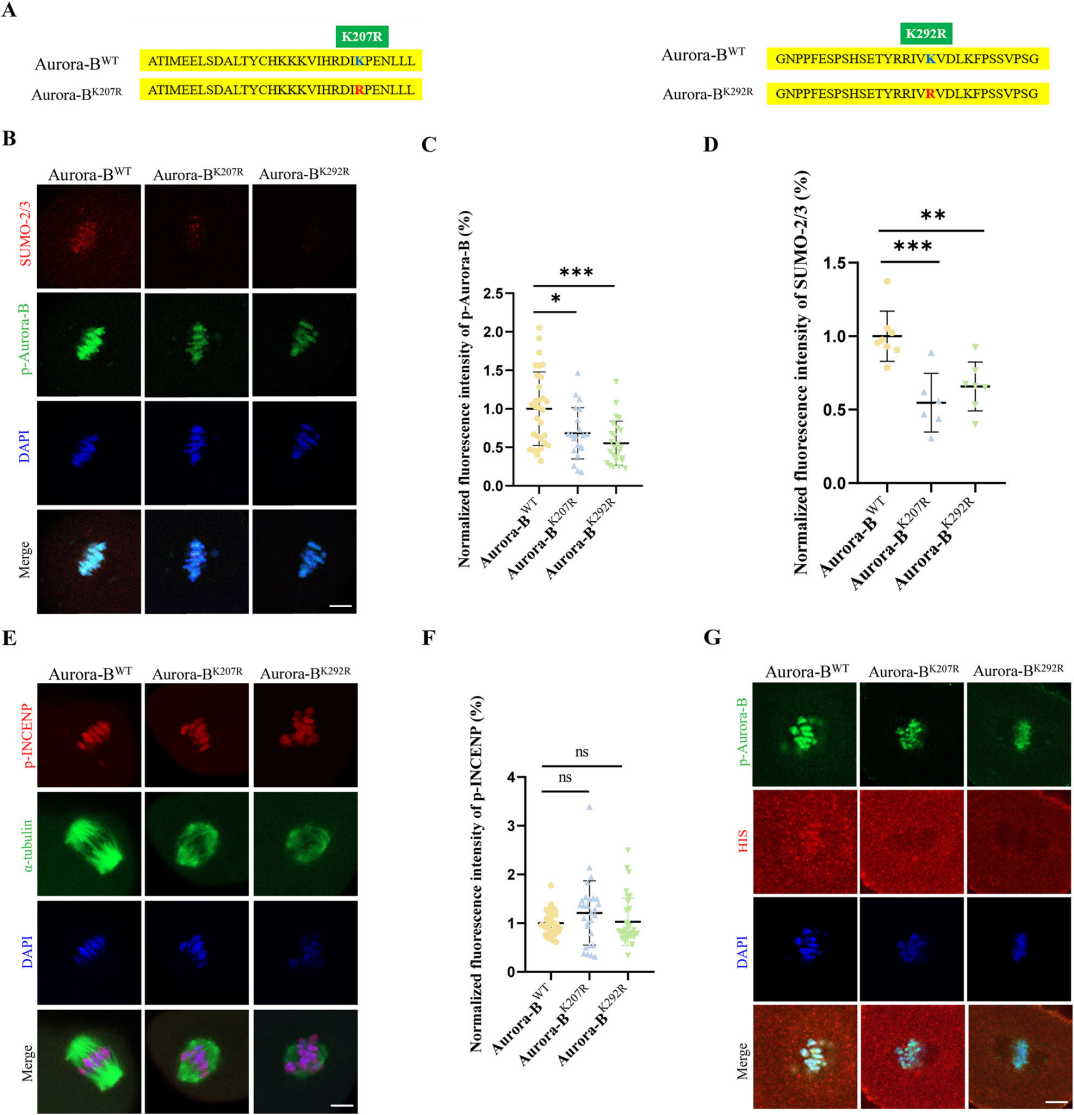
SUMOylation is essential for Aurora-B recruitment to mouse oocytes. **A** Synthetic Aurora-B^WT^, Aurora-B^K207R^, and Aurora-B^K292R^ mutant sequences. **B** Representative images of p-Auror-B and SUMO-2/3 in Aurora-B^WT^, Aurora-B^K207R^ and Aurora-B^K292R^ groups. SUMO-2/3 (red), p-Aurora-B (green), and DAPI-labeled DNA (blue) were used. Scale bars: 5 μm. **C** The fluorescence intensity of p-Aurora-B in MI oocytes of Aurora-B^WT^, Aurora-B^K207R^ and Aurora-B^K292R^ groups. *P < 0.05, ***P < 0.001. **D** The fluorescence intensity of SUMO-2/3 in MI oocytes of Aurora-B^WT^, Aurora-B^K207R^ and Aurora-B^K292R^ groups. **P < 0.01, ***P < 0.001. **E** Representative images of p-INCENP in MI oocytes from the Aurora-B^WT^, Aurora-B^K207R^ and Aurora-B^K292R^ groups. p-INCENP (red), α-tubulin (green) and DAPI-labeled DNA (blue). Scale bars: 5 μm. **F** The fluorescence intensity of p-INCENP in MI oocytes of Aurora-B^WT^, Aurora-B^K207R^ and Aurora-B^K292R^ groups. ns *P* > 0.05. (**G**) Representative images of His-tag in MI oocytes from the Aurora-B^WT^, Aurora-B^K207R^ and Aurora-B^K292R^ groups. His-tag (red), p-Aurora-B (green) and DAPI-labeled DNA (blue). Scale bars: 5 μm.

SUMOylated site mutants of Aurora-B were employed to evaluate the function of SUMOylated Aurora-B in oocytes. Aurora-B^K207R^ and Aurora-B^K292R^ was predicted by SUMOylation site (SUMOplot ™ Analysis Program http://www.abgent.com/tools) basing on mouse Aurora-B protein sequence (NP_035626.1), and it has been confirmed that Lys‑207 site was SUMOylated in mitosis [34, 35, 37]. Instead of traditional inhibitors, the SUMOylation site mutants, Aurora-B^K207R^ and Aurora-B^K292R^, were used to further explore the effect of Aurora-B SUMOylation, which avoids the side effects of other SUMOylated proteins (Fig. 3A). The average fluorescence intensity of phosphorylated Aurora-B in oocytes of two mutant groups, Aurora-B^K207R^ and Aurora-B^K292R^, was significantly lower than that of Aurora-B^WT^ group (Aurora-B^WT^: 1 ± 0.4783, n = 31 vs Aurora-B^K207R^: 0.6818 ± 0.3321, n = 21, *P* < 0.05; Aurora-B^WT^: 1 ± 0.4783, n = 31, vs Aurora-B^K292R^: 0.5538 ± 0.2868, n = 25, *P* < 0.001) (Fig. 3B, C). The average fluorescence intensity of SUMO-2/3 in oocytes of two mutant groups, Aurora-B^K207R^ and Aurora-B^K292R^, was significantly lower than that of Aurora-B^WT^ group (Aurora-B^WT^: 1 ± 0.1710, n = 8 vs Aurora-B^K207R^: 0.5474 ± 0.1990, n = 6, *P* < 0.001; Aurora-B^WT^: 1 ± 0.1710, n = 8 vs Aurora-B^K292R^: 0.6582 ± 0.1659, n = 7, *P* < 0.01) (Fig. 3B, D). Then, p-INCENP, the binding sites for Aurora-B recruitment [38], was detected to evaluate whether the mutation of SUMOylated sites on Aurora-B impacts the binding sites of its recruitment. There was no significant difference in the fluorescence intensity of p-INCENP between Aurora-B^WT^ and the two mutant groups, Aurora-B^K207R^ and Aurora-B^K292R^ in MI stage oocytes (Aurora-B^WT^: 1 ± 0.2422, n = 37 vs Aurora-B^K207R^: 1.208 ± 0.6611, n = 28, *P* > 0.05; Aurora-B^WT^: 1 ± 0.2422, n = 37 vs Aurora-B^K292R^: 1.028 ± 0.4911, n = 33, *P* > 0.05) (Fig. 3E, F). The result excludes the impact of binding sites to the reduce of p-Aurora-B recruitment. His-tag, binding with injected Aurora-B mRNA, was detected to show exogenous Aurora-B. Compared with that in Aurora-B^WT^ group, the recruitment of the exogenous Aurora-B^K207R^ and Aurora-B^K292R^ groups decreased, and the proteins even disappeared from the chromosomes (Fig. 3G). In addition, it has been confirmed that Aurora-A is recruited on chromosomes to partially compensate for the depletion of Aurora-B on the chromosomes in oocytes [39, 40], so Aurora-A was analyzed in oocytes of Aurora-B mutant. The results showed that no Aurora-A was detected on chromosomes in the Aurora-B^K207R^ and Aurora-B^K292R^ cells, where intensity of Aurora-B was reduced (Fig. S1), suggesting that no compensation of Aurora-A was found in oocytes of Aurora-B mutant. These results indicated that SUMOylation is essential for Aurora-B recruitment in oocytes.

### SUMOylation of Aurora-B regulates spindle formation and chromosome alignment in mouse oocyte meiosis

**Fig. 4:**
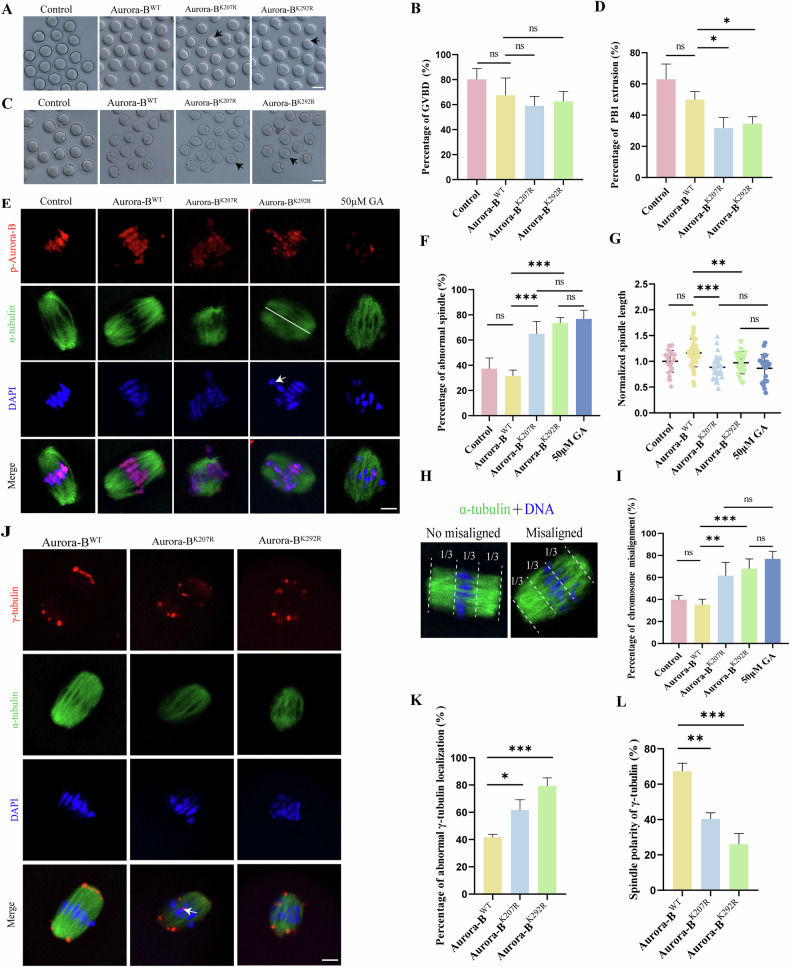
SUMOylation of Aurora-B regulates spindle formation and chromosome alignment in mouse oocyte meiosis. **A** Representative images of GVBD in mouse oocytes of control, Aurora-B^WT^, Aurora-B^K207R^ and Aurora-B^K292R^ groups. The black arrow indicates that the oocyte failed GVBD. Scale bars: 80 μm. **B** The percentage of GVBD after 4 h of culture in mouse oocytes of control, Aurora-B^WT^, Aurora-B^K207R^ and Aurora-B^K292R^ groups. ns *P* > 0.05. **C** Representative images of the first polar body extrusion in mouse oocytes of control, Aurora-B^WT^, Aurora-B^K207R^ and Aurora-B^K292R^ groups. The black arrow indicates that the oocyte failed to extrude the first polar body. Scale bars: 80 μm. **D** The percentage of first polar body extrusion after 13 h of culture in mouse oocytes of control, Aurora-B^WT^, Aurora-B^K207R^ and Aurora-B^K292R^ groups. ns *P* > 0.05, *P < 0.05. **E** Representative images of spindle morphology and chromosome alignment in MI oocytes of control, Aurora-B^WT^, Aurora-B^K207R^, Aurora-B^K292R^ and GA groups. p-Aurora-B (red), α-tubulin (green) and DAPI-labeled DNA (blue). White line showing the distance between two poles of spindle was measured as spindle length. White arrow shows misaligned chromosomes. Scale bars: 5 μm. **F** The percentage of abnormal spindle morphology in MI oocytes of control, Aurora-B^WT^, Aurora-B^K207R^, Aurora-B^K292R^ and GA groups. An oocyte without typical barrel-shaped spindle was marked as abnormal spindle morphology. ns *P* > 0.05, ***P < 0.001. **G** The percentage of spindle length in mouse oocytes of control, Aurora-B^WT^, Aurora-B^K207R^, Aurora-B^K292R^ and GA groups. ns *P* > 0.05, **P < 0.01 and ***P < 0.001. **H** An oocyte was defined as chromosome misalignment when a chromosome fails to locate in the central third of spindle. **I** The percentage of chromosome misalignment in MI oocytes of control, Aurora-B^WT^, Aurora-B^K207R^, Aurora-B^K292R^ and GA groups. ns *P* > 0.05, **P < 0.01 and ***P < 0.001. **J** Localization of γ‐tubulin in mouse oocytes of Aurora-B^WT^, Aurora-B^K207R^ and Aurora-B^K292R^ groups. γ‐tubulin (red), α-tubulin (green) and DAPI-labeled DNA (blue). White arrow indicates abnormal localization of γ‐tubulin. Scale bars: 5 μm. **K** The oocyte percentage of abnormal γ‐tubulin localization in MI oocytes of Aurora-B^WT^, Aurora-B^K207R^ and Aurora-B^K292R^ groups. An oocyte was defined as abnormal γ‐tubulin localization when all of or part of γ‐tubulin locate out of spindle poles. *P < 0.05, ***P < 0.001. **L** The percentage of spindle polarity of γ‐tubulin in MI oocytes of Aurora-B^WT^, Aurora-B^K207R^ and Aurora-B^K292R^ groups. The clusters of γ‐tubulin was counted, and the cluster number percentage of γ‐tubulin on spindle polar was calculated. **P < 0.01, ***P < 0.001.

The stage of meiosis when SUMOylated Aurora-B performs its function in oocytes was preliminarily estimated by analyses of the GVBD rate and first polar body extrusion (PB1) rate. Results showed that there was no significant difference in GVBD rate between control group and Aurora-B^WT^ group, and between Aurora-B^WT^ group and mutant groups (Aurora-B^K207R^ and Aurora-B^K292R^) (Control: 80.20 ± 8.701%, n = 139 vs Aurora-B^WT^: 67.60 ± 13.79%, n = 129, *P* > 0.05; Aurora-B^WT^: 67.60 ± 13.79%, n = 129 vs Aurora-B^K207R^: 59.00 ± 7.649%, n = 108, *P* > 0.05; Aurora-B^WT^: 67.60 ± 13.79%, n = 129 vs Aurora-B^K292R^: 62.60 ± 7.861%, n = 168, *P* > 0.05) (Fig. 4A, B). However, the percentage of PB1 extrusion decreased significantly in the Aurora-B^K207R^ and Aurora-B^K292R^ mutant groups compared with that of in Aurora-B^WT^ group, while there was no significant difference in the PB1 rate between control group and Aurora-B^WT^ group (Control: 63.00 ± 9.764%, n = 123 vs Aurora-B^WT^: 50.00 ± 5.099%, n = 106, *P* > 0.05; Aurora-B^WT^: 50.00 ± 5.099%, n = 106 vs Aurora-B^K207R^: 31.75 ± 6.752%, n = 92, *P* < 0.05; Aurora-B^WT^: 50.00 ± 5.099%, n = 106 vs Aurora-B^K292R^: 34.50 ± 4.509%, n = 113, *P* < 0.05) (Fig. 4C, D). These results showed that the mutated SUMOylation site of Aurora-B had no significant effect on the GVBD but significantly inhibited PB1 extrusion in mouse oocytes.

Then, SUMO conjugation site mutants of Aurora-B and SUMOylation inhibitor (50 μM GA) were used to assess their impact on spindle morphology in MI oocytes. We found that overexpression of both Aurora-B^K207R^ and Aurora-B^K292R^ clearly disrupted spindle organization. Oocytes in control group and Aurora-B^WT^ group had typical barrel-shaped spindles and accurate alignment of chromosomes in MI oocytes. However, the Aurora-B^K207R^, Aurora-B^K292R^ and GA groups had abnormal spindle morphology and misaligned chromosomes (Fig. 4E). The statistical results showed that there was no significant difference in abnormal spindles between control group and Aurora-B^WT^ group (Control: 37.25 ± 8.500%, n = 60 vs Aurora-B^WT^: 31.50 ± 4.726%, n = 71, *P* > 0.05). The percentage of abnormal spindles in Aurora-B^K207R^ and Aurora-B^K292R^ mutant groups was significantly greater than that of in Aurora-B^WT^ group (Aurora-B^WT^: 31.50 ± 4.726%, n = 71 vs Aurora-B^K207R^: 65.00 ± 9.764%, n = 66, *P* < 0.001; Aurora-B^WT^: 31.50 ± 4.726%, n = 71 vs Aurora-B^K292R^: 73.50 ± 4.435%, n = 54, *P* < 0.001), while no significant difference with in that of GA group (Aurora-B^K207R^: 65.00 ± 9.764%, n = 66 vs GA: 76.75 ± 6.946%, n = 49, *P* > 0.05; Aurora-B^K292R^: 73.50 ± 4.435%, n = 54 vs GA: 76.75 ± 6.946%, n = 49, *P* > 0.05) (Fig. 4F). The statistical results of spindle length showed that there was no significant difference of spindle length in control group and Aurora-B^WT^ group (Control: 1 ± 0.2122, n = 28 vs Aurora-B^WT^: 1.163 ± 0.2713, n = 36, *P* > 0.05). The average spindle length in the Aurora-B^K207R^ and Aurora-B^K292R^ mutant groups was significantly shorter comparing to those of in Aurora-B^WT^ group (Aurora-B^WT^: 1.163 ± 0.2713, n = 28 vs Aurora-B^K207R^: 0.8846 ± 0.2150, n = 33, *P* < 0.001; Aurora-B^WT^: 1.163 ± 0.2713, n = 28 vs Aurora-B^K292R^: 0.9712 ± 0.2141, n = 32, *P* < 0.01), and the spindle length have no significant difference in those of two mutant groups and GA group (Aurora-B^K207R^: 0.8846 ± 0.2150, n = 33 vs GA: 0.8647 ± 0.2689, n = 22, *P* > 0.05; Aurora-B^K292R^: 0.9712 ± 0.2141, n = 32 vs GA: 0.8647 ± 0.2689, n = 22, *P* > 0.05) (Fig. 4G). The chromosomes of oocytes in the Aurora-B^K207R^ and Aurora-B^K292R^ groups were severely misaligned (An oocyte was defined as chromosome misalignment when a chromosome fails to locate in the central third of spindle [41]) (Fig. 4H). The statistical results of misaligned chromosomes showed there was no significant difference of misaligned chromosomes between control group and Aurora-B^WT^ group (Control: 39.75 ± 3.948%, n = 60 vs Aurora-B^WT^: 35.25 ± 4.992%, n = 71, *P* > 0.05). The percentages of misaligned chromosomes in the Aurora-B^K207R^ and Aurora-B^K292R^ groups were significantly greater than those of in Aurora-B^WT^ group (Aurora-B^WT^: 35.25 ± 4.992%, n = 71 vs Aurora-B^K207R^: 61.50 ± 12.07%, n = 66, *P* < 0.01; Aurora-B^WT^: 35.25 ± 4.992%, n=71 vs Aurora-B^K292R^: 68.00 ± 8.756%, n = 54, *P* < 0.001), and no significant difference with GA group (Aurora-B^K207R^: 61.50 ± 12.07%, n = 66 vs GA: 76.75 ± 6.946%, n = 49, *P* > 0.05; Aurora-B^K292R^: 68.00 ± 8.756%, n = 54 vs GA: 76.75 ± 6.946%, n = 49, *P* > 0.05) (Fig. 4I).

Gamma-tubulin (γ‐tubulin), a key member of microtubule nucleation for microtubule organizing center (MTOCs) formation [42], was employed to show the stability of spindle poles. We found that γ‐tubulin was localized only at spindle poles in MI oocytes in Aurora-B^WT^ group (Fig. 4J); however, part of γ‐tubulin was observed to localize out of spindle poles in Aurora-B^K207R^ and Aurora-B^K292R^ groups (Fig. 4J). The statistical analysis showed that the abnormal oocyte percentage of γ‐tubulin localization in the Aurora-B^K207R^ and Aurora-B^K292R^ groups was significantly greater than that of in Aurora-B^WT^ group (Aurora-B^WT^: 41.67 ± 2.082%, n = 58 vs Aurora-B^K207R^: 61.67 ± 7.572%, n = 57, *P* < 0.05; Aurora-B^WT^: 41.67 ± 2.082%, n = 58 vs Aurora-B^K292R^: 79.33 ± 5.859%, n = 40, *P* < 0.001) (Fig. 4K). Furthermore, the percentages of spindle polarity of γ‐tubulin in the Aurora-B^K207R^ and Aurora-B^K292R^ groups were significantly lower than that of in Aurora-B^WT^ group (Aurora-B^WT^: 67.33 ± 4.509%, n = 190 vs Aurora-B^K207R^: 40.33 ± 3.512, n = 173, *P* < 0.01; Aurora-B^WT^: 67.33 ± 4.509%, n = 190 vs Aurora-B^K292R^: 29.33 ± 3.512%, n = 142, *P* < 0.001) (Fig. 4L). Meanwhile, there was no significant difference in the average fluorescence intensity of γ‐tubulin between Aurora-B^WT^ group and above two mutant groups (Aurora-B^WT^: 1 ± 0.3365, n = 37 vs Aurora-B^K207R^: 1.184 ± 0.4588, n = 28, *P* > 0.05; Aurora-B^WT^: 1 ± 0.3365, n = 37 vs Aurora-B^K292R^: 1.164 ± 0.4590, n = 27, *P* > 0.05) (Fig. S2). These results indicated that SUMOylation of Aurora-B plays a crucial role in spindle organization and chromosome alignment in mouse oocytes.

### SUMOylation of Aurora-B regulates tubulin acetylation in mouse oocytes

**Fig. 5:**
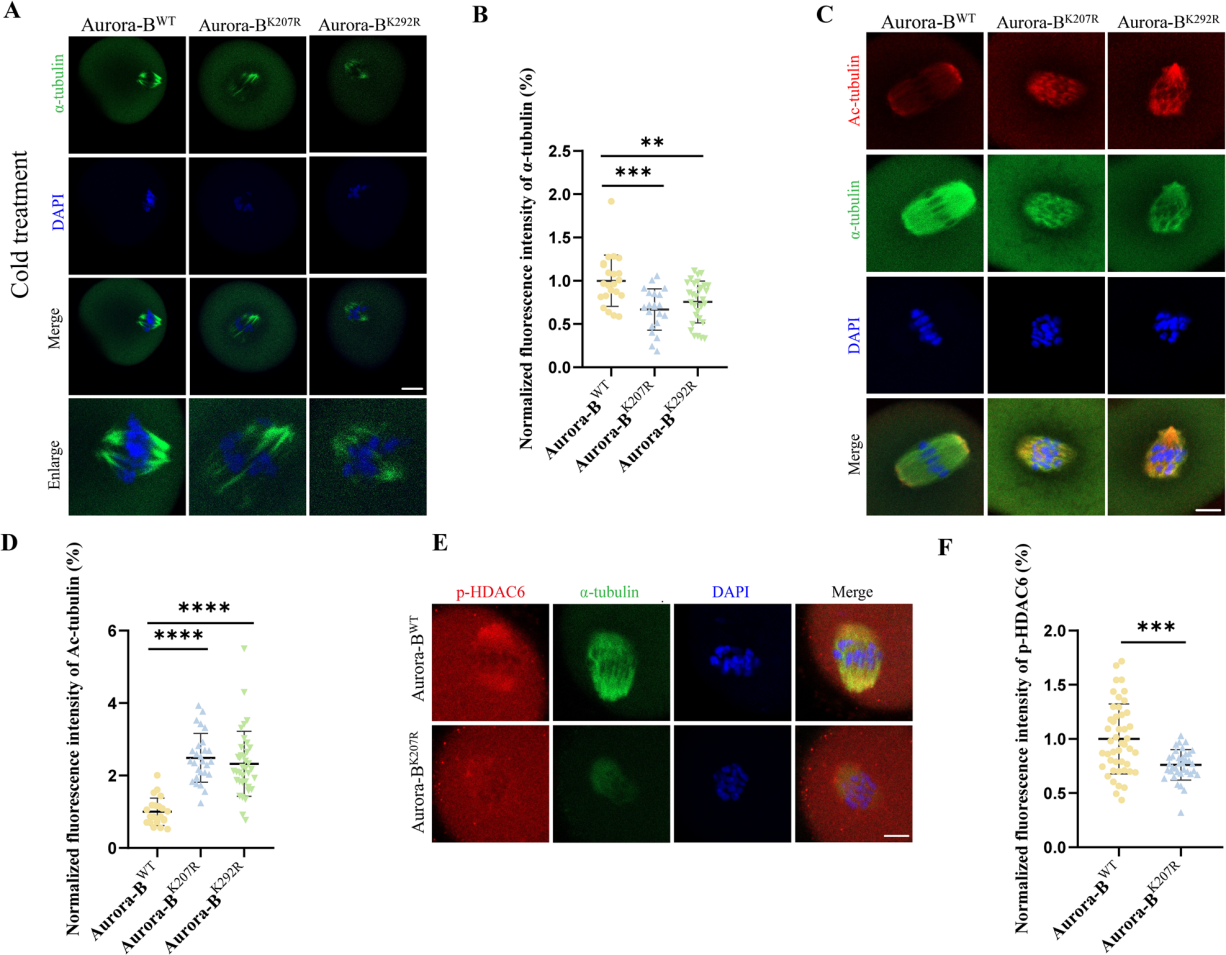
SUMOylation of Aurora-B regulates tubulin acetylation levels in mouse oocytes. **A** Representative images of α-tubulin in MI oocytes of Aurora-B^WT^, Aurora-B^K207R^ and Aurora-B^K292R^ groups after cold treatment. α-tubulin (green) and DAPI-labeled DNA (blue). Scale bar: 20 μm. **B** The fluorescence intensity of α-tubulin in MI oocytes in Aurora-B^WT^, Aurora-B^K207R^ and Aurora-B^K292R^ groups. **P < 0.01, ***P < 0.001. **C** Representative images of acetylated α-tubulin in MI oocytes of Aurora-B^WT^, Aurora-B^K207R^ and Aurora-B^K292R^ groups. Ac-tubulin (red), α-tubulin (green) and DAPI-labeled DNA (blue). Scale bar: 5 μm. **D** The fluorescence intensity of ac-tubulin in MI oocytes of Aurora-B^WT^, Aurora-B^K207R^ and Aurora-B^K292R^ groups. ****P < 0.0001. **E** Representative images of p-HDAC6 in MI oocytes of Aurora-B^WT^, Aurora-B^K207R^ and Aurora-B^K292R^ groups. p-HDAC6 (red), α-tubulin (green) and DAPI-labeled DNA (blue). Scale bar: 5 μm. **F** The fluorescence intensity of p-HDAC6 signals in MI oocytes of Aurora-B^WT^ and Aurora-B^K207R^ groups. ***P < 0.001.

To further explore the impact of SUMOylation of Aurora-B on microtubule dynamics, cold treatment was employed to depolymerize the unattached microtubules, which was followed by measurement of α-tubulin intensity as a proxy of correct attached microtubule [41]. Statistical analysis revealed that the fluorescence intensities of α-tubulin in Aurora-B^K207R^ and Aurora-B^K292R^ groups were significantly lower than that of in Aurora-B^WT^ group (Aurora-B^WT^: 1 ± 0.2966, n = 22 vs Aurora-B^K207R^: 0.6681 ± 0.2401, n = 21, *P* < 0.001; Aurora-B^WT^: 1 ± 0.2966, n = 22 vs Aurora-B^K292R^: 0.7553 ± 0.2411, n = 30, *P* < 0.01) (Fig. 5A, B), suggesting the reduce of correct attached stable microtubule. To evaluate the stability of whole spindle microtubules, we detected tubulin acetylation levels through immunostaining. Statistical analysis revealed that the fluorescence intensities of ac-tubulin in Aurora-B^K207R^ and Aurora-B^K292R^ groups were significantly higher than that of in Aurora-B^WT^ group (Aurora-B^WT^: 1 ± 0.3778, n = 22 vs Aurora-B^K207R^: 2.489 ± 0.6729, n = 27, *P* < 0.0001; Aurora-B^WT^: 1 ± 0.3778, n = 22 vs Aurora-B^K292R^: 2.325 ± 0.8990, n = 37, *P* < 0.0001) (Fig. 5C, D). Histone deacetylases-6 (HDAC6), an important enzyme regulating the acetylation level of tubulin [43] was employed to reveal the regulation of tubulin acetylation. The results showed that, compared with those in Aurora-B^WT^ group, the fluorescence signals of p-HDAC6 were decreased in the Aurora-B^K207R^ group (Aurora-B^WT^: 1 ± 0.3222, n = 46 vs Aurora-B^K207R^: 0.7606 ± 0.1403, n = 36, *P* < 0.001) (Fig. 5E, F). Collectively, these results suggested that SUMOylation of Aurora-B modulated the stability of microtubules at the tubulin acetylation level, that is important for spindle formation in oocytes.

## Discussion

Previous studies have shown that Aurora-B is a SUMO2/3 conjugation substrate in mitosis [34, 36, 44, 45]. Aurora-B plays a role in regulating chromosome arrangement and separation, cytoplasmic division, and microtubule dynamics in many more organisms [24, 25, 46, 47]. Previous studies found that SUMOylation regulates oocyte meiosis [15]. In the present study, we reported that SUMOylation is involved in the control of Aurora-B function, which is important for meiotic spindle formation during oocyte meiosis. Previous studies showed that Aurora-B was localized to the nucleus of oocytes at GV stage of oocytes, was enriched in chromatin regions at GVBD stage [24, 48, 49], was localized to chromosomes at MI stage and was enriched in centromeres and the spindle at MII stage [24, 48–50]. These are consistent with the results of our study. However, until now, there has been no evidence for a relationship between SUMOylation and the function of Aurora-B in oocyte meiosis. In this study, we showed the colocalization of Aurora-B and SUMO-2/3 in pre-MI, MI and MII oocytes during meiosis, suggesting the possibility of an interaction between Aurora-B and SUMO-2/3. Then, immunoprecipitation experiments were performed and indicated that Aurora-B could be modified by SUMOylation during oocyte meiosis. However, not all of Aurora-B protein is modified by SUMO2/3, take Aurora-B in midzone without colocalization with SUMO-2/3 in AI oocytes for example (Fig. 1A), which suggests that Aurora-B also could be modified by other SUMO proteins even other pathway of Protein post-translational modification in oocytes. In addition, AMPK, belonging the same serine/threonine protein kinase with Aurora-B, decreased in oocytes of aged mice [51]. So, the correlation of Aurora-B and aging was detected. But we found that the level of the phosphorylated Aurora-B protein was not significantly related to maternal age. However, when maternal age increased, the SUMO-2/3 protein in oocytes decreased significantly, which was consistent with earlier studies on the link between age and SUMOylation modification in mouse oocytes [10]. This result indicated that SUMOylation may be related to maternal aging, but not through modulation of Aurora-B activity.

To verify the hypothesis that Aurora-B is modified by SUMOylation and to define molecular mechanism of this interaction, we constructed two SUMOylated Aurora-B site mutants in which Lys K was mutated to Arg R, preventing the mutated site from being able to undergo SUMOylation, rather than traditional inhibitors to avoid the negative effects on other SUMOylated proteins. Previous studies have shown that SUMO-2 can preferentially modify the Lys202 residue of Aurora-B, promoting self-phosphorylation of Aurora-B in human cells [34]. Aurora-B may be preferentially modified by SUMO-2 and SUMO-3, and the Lys-207 residue of the Aurora-B mutant results in abnormal spindle morphology and disrupted chromosome organization in HEK293 cells [44]. In the ovaries of mice, Lys-207 of the Aurora-B site is a major modification site, and SUMOylation modulates follicular development through an increase in Aurora-B localization in the nucleus [35]. However, it is still unclear whether the Lys-207 residue plays an important role in the SUMOylation of Aurora-B in MI oocytes. We also constructed another site, Lys-292, to further verify its regulatory mechanism in oocyte meiosis. We found that the mutation of SUMOylation sites of Aurora-B affected the recruitment of Aurora-B and reduced the phosphorylation level of Aurora-B after the injection of exogenous Aurora-B, and fluorescence staining with a His-tag confirmed this finding. We confirmed the Lys-207 [35] and Lys-292 are major modification sites in mouse oocytes, and the Lys-292 site is more likely to cause a decrease in Aurora-B phosphorylation levels and a decrease in SUMO-2/3 recruitment. However, Aurora-B SUMOylation did not affect the localization of INCENP, a scaffolding subunit of the CPC [38, 52]. The C-terminal IN-box structural domain of INCENP was shown to specifically bind to and enhance the activity of Aurora-B [53]. A previous study showed that prevention of SUMOylation by the usage of Aurora-B^K207R^ mutant disrupted the localization of Aurora-B and INCENP in prometaphase and metaphase during mitosis [44]. It indicates that the function of SUMOylated Aurora-B may differ in the impact of INCENP recruitment between mitosis and meiosis. On the other hand, previous studies revealed that when Aurora-B/C was knocked out in mouse oocytes, a portion of Aurora-A departed from the spindle pole and relocated to the chromosome to compensate for the deletion of Aurora-B [39]. But Aurora-A that is essential for meiosis in mouse oocytes cannot be compensated by Aurora-B/C [40]. In this study, results show that no localization of Aurora-A on chromosomes was found in mutational oocytes of Aurora-B, suggesting that the decrease of Aurora-B recruitment contributing by SUMOylation sites mutation cannot be rescued by Aurora-A.

Next, we explored the effects of Aurora-B SUMOylation on oocyte maturation. Previous studies showed that the inhibition of Aurora-B/C during meiotic maturation resulted in chromosome segregation errors and decreased the percentage of PB1 extrusion [54, 55]. Here, we found that overexpressing SUMOylated Aurora-B site mutants did not affect the process of GVBD but greatly inhibited PB1 extrusion in mouse oocytes. In addition, the results of overexpressing Aurora-B^122R^, a kinase dominant-negative Aurora-B mutant, in vertebrate oocytes suggest that Aurora-B regulates spindle bipolarity by inhibiting MCAK [56]. Mutation of the SUMOylation site Aurora-B^K207R^ caused abnormal spindle morphology and chromosome misalignment during mitosis [44]. We showed that SUMOylation of Aurora-B site mutants disrupted meiotic spindle formation and chromosome alignment, which was confirmed by the aberrant localization of γ‐tubulin, a molecule localized on the pole of the spindle that facilitates microtubule nucleation and assembly [42, 57]. Altogether, the experiment results for SUMOylated sites mutation of Aurora-B in oocytes in this study aligns with previous studies on block the function of Aurora-B [25, 26, 58]. It shows that the SUMOylation of Aurora-B is necessary for spindle assembly and PBE during meiosis of oocytes.

Previous studies reveal that Aurora-B is recruited by incorrect KT-MT attachments and correct them by reducing microtubule stability [59]. Therefore, the role of SUMOylation of Aurora-B on microtubule stability is analyzed in this study. For cold treatment to depolymerize unattached microtubules [41], the results show the correctly attached stable microtubules reduce in SUMOylation site mutant oocytes of Aurora-B. On this basis, the stability of microtubules was analyzed further in above mutant oocytes. Tubulin acetylation is related to maintenance of stable microtubules [60–62]. Inhibiting Kinesin family member 11 (KIF11) in pig oocytes resulted in decreased acetylation of microtubule and weakened microtubule stability [63], indicating that microtubule acetylation could enhance microtubule stability in porcine oocytes. The acetylation level of tubulin was mainly regulated by histone acetyltransferase and histone deacetylase, including phosphorylated Histone deacetylase 6 (p-HDAC6), an α-tubulin deacetylase [60–62]. Our findings reveal that the SUMOylation site mutation of Aurora-B inhibits the recruitment and activation of HDAC6 on spindle microtubules, which causes the high stability of whole spindle microtubules in oocytes, including wrongly attatched microtubules. It further induces that more wrong KT-MT attachments, less correct ones through the failure of microtubule depolymerization of wrong KT-MT attachments, which contributes to abnormal spindle morphology and chromosome aneuploidy.

Besides correcting the KT-MT attachment, Aurora-B contributes on SAC in mitosis [22]. But some studies showed that SAC is not decreased in oocytes of Aurora-B inhibition [27] suggesting that SAC activation does not require Aurora-B/C kinase activity in oocytes. On the other hand, Aurora-B cKO studies [26] showed that nearly 100% of oocytes from young cKOs arrested at MI when cultured in nocodazole, showing a functional SAC in Aurora-B cKOs [22]. Both inhibitor and cKO studies have demonstrated clearly that SAC activation does not require Aurora-B/C kinase in oocytes from young mice. Interestingly, a dysfunctional SAC response is found in aged rather than young Aurora-B cKOs, suggesting that the deficiency of Aurora-B may contibute to rapid compromise of SAC integrity with maternal age. The function of SUMOylated Aurora-B on SAC integrity with maternal age is worth researching in future. Besides, it is observed that Aurora-B mutants induce the prolongation of MI stage in oocytes (Fig. 4G), but the downstream molecules of Aurora-B are still murky. Furthermore, it has been found that SUMOylation modification is involved in the recruitment of Aurora-B downstream factors, such as CPC, kinetochore motor CENP-E, etc., finally regulating chromosome alignment and spindle maintenance [33, 44, 64]. But it is still unclear that SUMOylation perform this function by modifying either Aurora-B or downstream factors.

## Conclusions

**Fig. 6:**
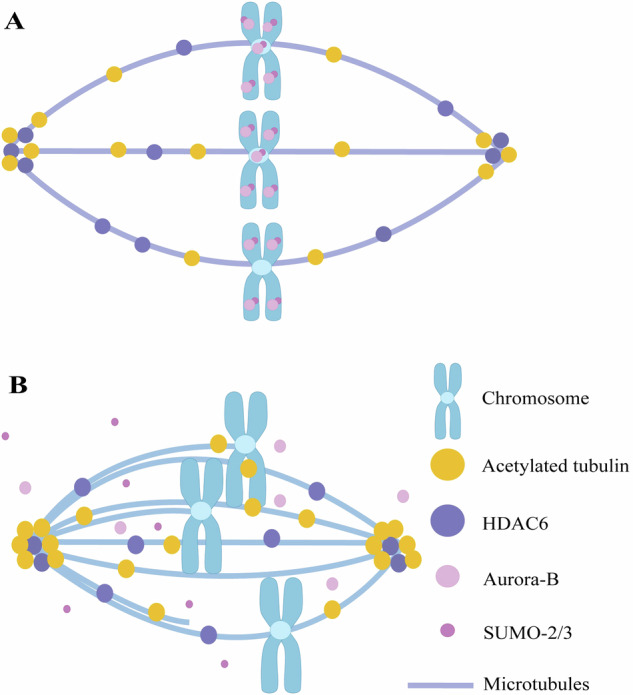
The roles of SUMOylated Aurora-B in oocyte spindle assembly. **A** SUMOylated Aurora-B is localized on chromosomes. **B** In oocytes in which the SUMOylation site of Aurora-B is mutated, Aurora-B dissociates from chromosomes, increasing the stability of acetylated microtubules while decreasing HDAC6 recruitment. This leads to chromosome misalignment and abnormal in spindle morphology.

In summary, we have shown that Aurora-B undergoes SUMOylated modification mediated by SUMO-2/3 during oocyte meiosis, and that mutating the SUMOylation site of Aurora-B disrupts spindle formation and chromosome alignment, which may contribute to defects in oocyte maturation during aging (Fig. 6).

## Materials and methods

### Antibodies and chemicals

Rabbit polyclonal antibody to phospho-Aurora-B (Thr232) (AF3475, for Fluorescence (IF) 1:200), and a rabbit polyclonal antibody to phospho-INCENP (Thr59) (AF8485, for IF 1:100) were obtained from Affinity. Rat monoclonal anti-SUMO-2 + SUMO-3 antibody [3H12] (GTX00699, for IF 1:100) was obtained from GeneTex. Rabbit monoclonal anti-gamma tubulin antibody [EPR16793] (ab179503, for IF 1:300) and Alexa Fluor 488 mouse monoclonal anti-alpha tubulin antibody [DM1A] (ab195887, for IF 1:300) were obtained from Abcam. Mouse monoclonal anti-acetylated tubulin antibody (T7451, for IF 1:300) was obtained from Sigma (St. Louis, MO, USA). Rabbit monoclonal anti-Aurora A antibody (AZ050, for IF 1:100) and mouse monoclonal anti-Tubulin antibody (AT819, for western blot (WB) 1:1000) were obtained from Beyotime. Alexa Fluor 594-conjugated goat anti-mouse IgG (ZF-0513, for IF, 1:200) was obtained from Zhongshan Golden Bridge Biotechnology (Beijing, China). Alexa Fluor 594-conjugated goat anti-rabbit IgG (H + L) (A11012, for IF 1:200) was obtained from ABclonal. The iFluorTM 488-conjugated goat anti-rabbit IgG polyclonal antibody (HA1121, for IF 1:200) was obtained from HUABIO Biotechnology (Hangzhou, China).

### Oocyte collection and culture in vitro

Mouse care and handing were conducted in accordance with the instructions of the Animal Ethics Committee of Zunyi Medical University and the Animal Ethics Committee of Nanjing Agricultural University. Female ICR mice (animal licence: SCXK 2021-0086, Henan SKBES Biotechnology, China) were used in this study. ICR mice, 6 weeks for the young group and 12 months for the old group, were euthanized 48 h after the injection of pregnant mare serum gonadotropin (PMSG, 5 IU per mouse). Ovaries were collected and chopped to release germinal vesicle (GV) oocytes. Oocytes with obvious germinal vesicles were picked and cultured to a designated stage in M2 medium (Sigma) at 37 °C with 5% CO_2_ for subsequent analysis. Generally, mouse oocytes in the GV stage were cultured for 6 h to the premetaphase (Pre-MI) stage, 8 h to the metaphase (MI) stage, 10 h to the anaphase (AI) stage, and 16 h to the metaphase II (MII) stage.

### Cold treatment

All the MI-stage oocytes were frozen at 4 °C for 10 min to depolymerize the microtubules. Cold treatment enabled clear observation of stable microtubules and kinetochore-microtubule attachments.

### Immunofluorescence staining

Oocytes from each group were fixed in 4% paraformaldehyde (Beyotime, P0099) for 30 min at room temperature and permeabilized in enhanced immunostaining permeabilization buffer (Beyotime, P0097) at room temperature for 30 min. Then, the oocytes were blocked in Immunol Staining Blocking Buffer (Beyotime, P0102) for at least 1 h at room temperature. The oocytes were incubated with primary antibodies (anti-p-Aurora-B, 1:200; anti-SUMO-2 + SUMO-3, 1:100; anti-His-tag, 1:50; anti-acetylated-tubulin, 1:300; anti-p-HDAC6, 1:100) at 4 °C overnight. The next day, the oocytes were incubated with the appropriate secondary antibodies (1:200) for 1 h at room temperature. Finally, the oocytes were mounted on glass slides with Antifade Mounting Medium with DAPI (Beyotime, P0131) and observed by a laser scanning confocal microscope (Zeiss LSM 800 META, Zena, Germany). After the above steps, the cells were washed with Immunol Staining Wash Buffer (Beyotime, P0106) 3 times for 2 min each.

### Plasmid construction and mRNA synthesis

Guangzhou Dahong Biotechnology Co., Ltd. synthesized the wild-type Aurora-B^WT^ and Aurora-B^K207R^ and Aurora-B^K292R^ gene sequences and cloned them into the target vector pcDNA3.1 (+) (ampicillin) with a His-tag. We verified the results by sequencing and synthesizing mRNA from the plasmid transcribed by using a HiScribe T7 High Yield RNA Synthesis Kit (NEB, E2040S) and capping it with m7G (5′) ppp (5′) G (NEB, S1404S) and *E. coli* poly (A) polymerase added to the poly(A) tail (Beyotime, R7070s). Finally, the mRNA was purified with RNA Clean & Concentrator (Zymo Research) and stored at −80 °C. GV oocytes were randomly and equally divided into each group, and 5-10 pL of Aurora-B^WT^ mRNA, mutant Aurora-B^K207R^ mRNA and Aurora-B^K292R^ mRNA (injection concentration: 500 ng/μL) were injected into oocytes of each group.

### Co-immunoprecipitation and Western blotting

For the Co-immunoprecipitation (Co-IP) assay, 1000 mouse oocytes were collected and washed twice with PBS. Then, the cells were lysed on ice for 30 min with RIPA lysis buffer containing proteinase inhibitor (Solarbio, R0010). Rabbit anti-Aurora-B polyclonal antibody was incubated overnight with protein A/G magnetic beads (MCE) and incubated with the cell lysate for 4 h at 4 °C. Then, we placed the tube on the magnet. Next, we washed the immune complexes three times and released them from the beads by mixing them in SDS loading buffer for 10 min at 30 °C. Subsequently, we supplemented the sample with SDS‒PAGE loading buffer and heated it at 100 °C for 10 min, after which western blotting was performed.

For western blotting, 150 mouse oocytes were collected and washed twice with PBS. Then, the cells were lysed on ice for 30 min with RIPA lysis buffer containing proteinase inhibitor (Solarbio, R0010). After adding SDS‒PAGE loading buffer (Solarbio, P1016), the samples were denatured at 100 °C for 10 min. After being subjected to 10% SDS‒PAGE, the proteins were transferred to a PVDF membrane and blocked with TBST containing 5% nonfat milk for 1 h at room temperature. The membranes were incubated with the following primary antibodies at 4 °C overnight: mouse monoclonal anti-Tubulin (1:1000), rabbit polyclonal anti-SUMO-2/3 (1:1000, Proteintech, 11251-AP), and rabbit polyclonal anti-Aurora-B (1:1000, Thermo Fisher). Then, the membranes were washed three times with TBST for 10 min and incubated at 37 °C for 1 h at room temperature with secondary antibody (1:8000). After washing three times with TBST for 10 min, we added developer and exposed the proteins using a fully automated chemiluminescence image analysis system.

### Statistical analysis

The sample size is chosen by SPSSAU (Power = 0.8). The labels of group name are covered by opaque stickers during detection. Unless indicated otherwise, all experiments are repeated three times. The fluorescence intensities of all groups were measured in the same batch under the same staining procedure, confocal microscope and parameter. Images presented from microscopy are maximum intensity projections of multiple acquired planes. Data out of 3σ is defined into abnormal data and is removed in data preparation. The data were analyzed using GraphPad Prism 8 (GraphPad Software, USA) and ImageJ software for band grayscale analysis and fluorescence intensity analysis. The statistical significance of the young and aged groups was compared using unpaired two-tailed Student’s t-test, whereas the control, Aurora-B^WT^ group, Aurora-B^K207R^ and Aurora-B^K292R^ groups were compared using one-way ANOVA. The data were presented as mean ± standard error (mean± SD). *P* < 0.05 was considered statistically significant.

## Supplementary information

Legends of supplemental figure

Supplemental figure 1

Supplemental figure 2

Supplemental figure 3

## Data Availability

The data used and analyzed during the current research period are available from the corresponding authors upon reasonable request.
