# Supplementary material for: Mutation of the SUMOylation site of Aurora-B disrupts spindle formation and chromosome alignment in oocytes
Source: Cell Death Discov. 2024 Oct 22;10:447. doi: 10.1038/s41420-024-02217-7 (PMC11496499; doi:10.1038/s41420-024-02217-7)
Supplement: Supplementary file 1 — Legends of supplemental figure [file 41420_2024_2217_MOESM1_ESM.docx]

**Legends of supplemental figure**

**Supplemental figure 1.** Representative images of Aurora-A in MI oocytes of Aurora-B^WT^, Aurora-B^K207R^ and Aurora-B^K292R^ groups. Aurora-A (red), α-tubulin (green) and DAPI-labeled DNA (blue). Scale bars: 5 μm.

**Supplemental figure 2.** The fluorescence intensity of γ‐tubulin in MI oocytes of Aurora-B^WT^, Aurora-B^K207R^ and Aurora-B^K292R^ groups. ns *P*>0.05.

**Supplemental figure 3.** Original western blots.
