# Supplementary figures and images for: Mutation of the SUMOylation site of Aurora-B disrupts spindle formation and chromosome alignment in oocytes
Source: Cell Death Discov. 2024 Oct 22;10:447. doi: 10.1038/s41420-024-02217-7 (PMC11496499; doi:10.1038/s41420-024-02217-7)

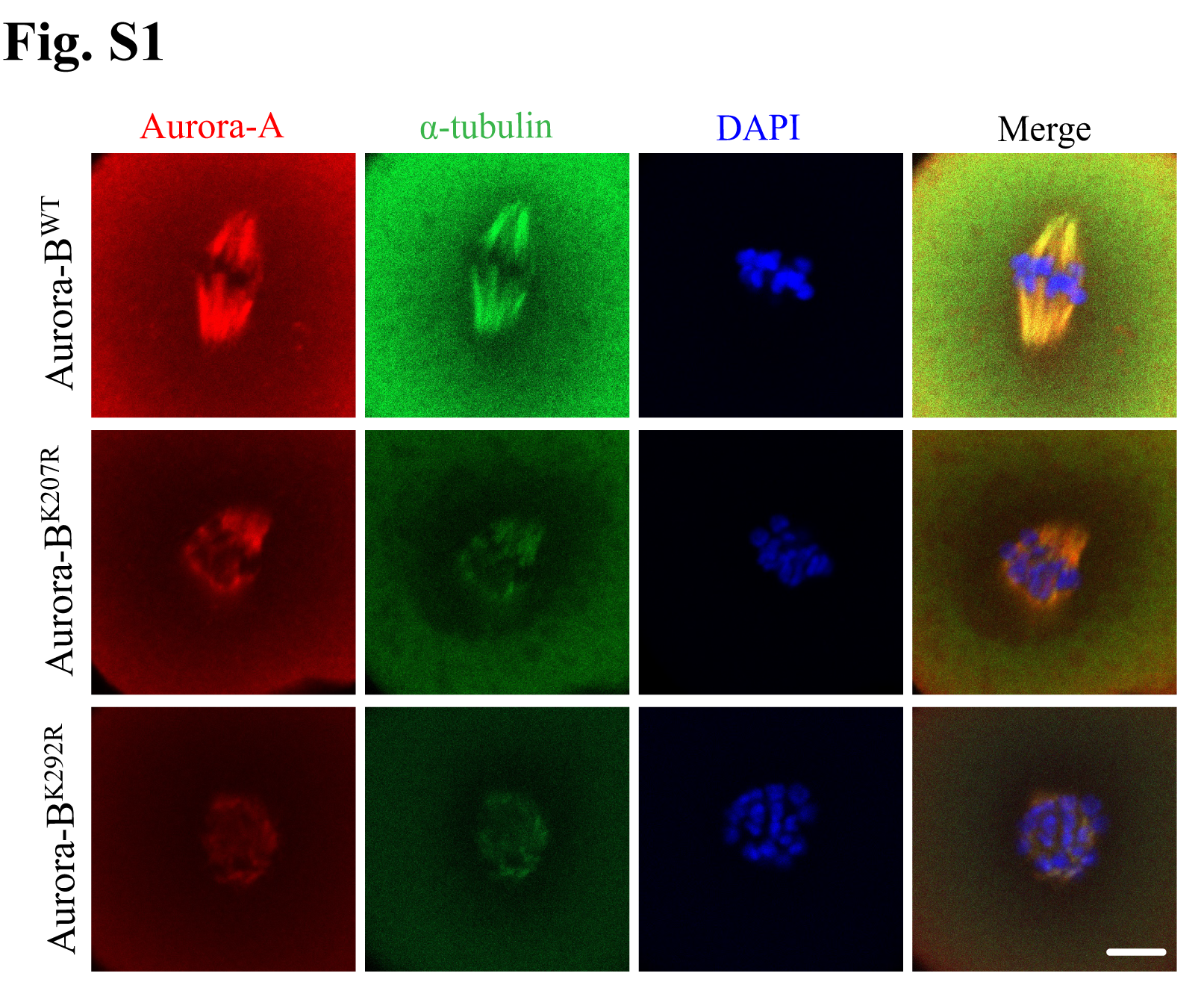

Supplement: Supplementary file 2 — Supplemental figure 1 [file 41420_2024_2217_MOESM2_ESM.tif]

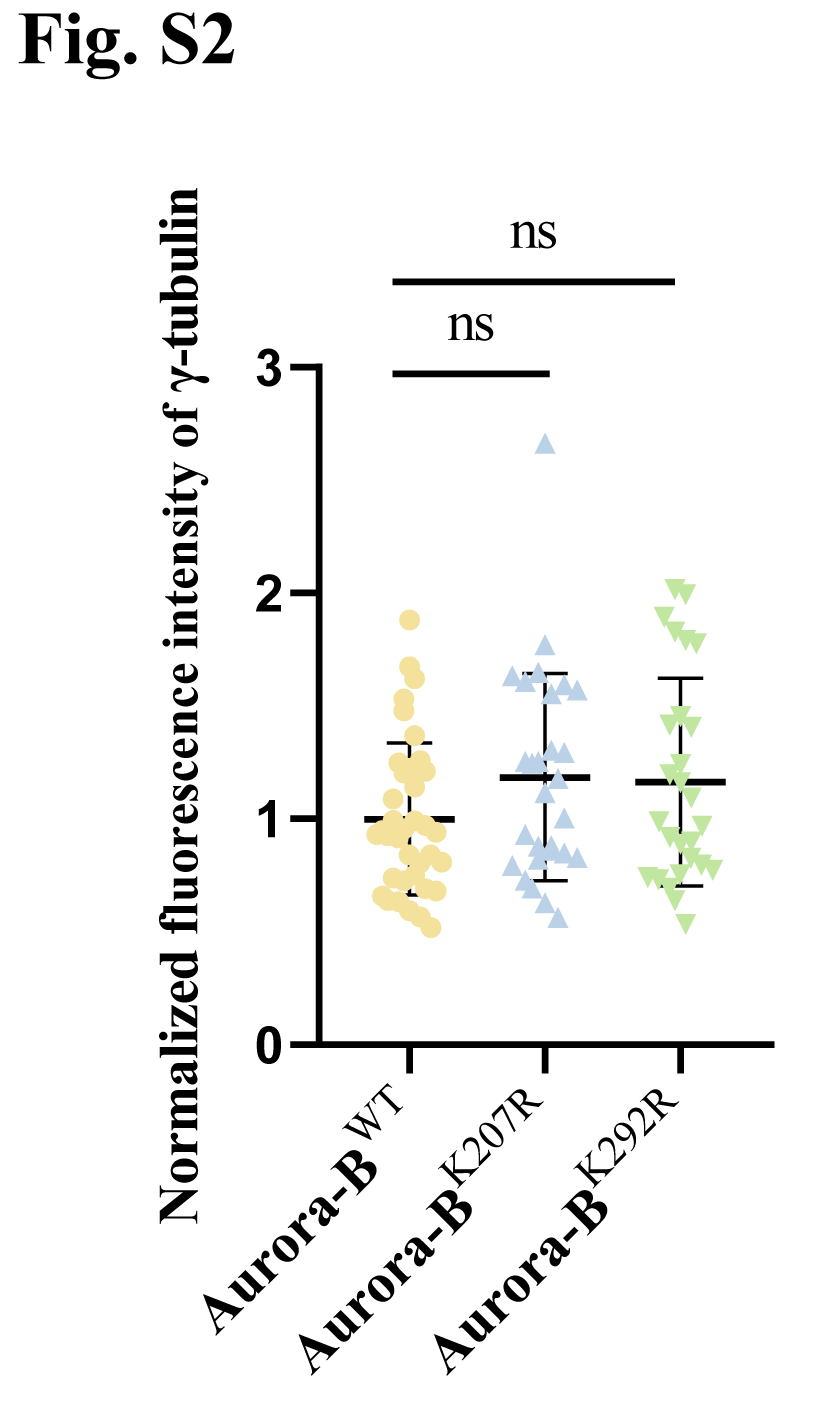

Supplement: Supplementary file 3 — Supplemental figure 2 [file 41420_2024_2217_MOESM3_ESM.tif]
