# Supplementary material for: Mutation of the SUMOylation site of Aurora-B disrupts spindle formation and chromosome alignment in oocytes
Source: Cell Death Discov. 2024 Oct 22;10:447. doi: 10.1038/s41420-024-02217-7 (PMC11496499; doi:10.1038/s41420-024-02217-7)
Supplement: Supplementary file 4 — Supplemental figure 3 [file 41420_2024_2217_MOESM4_ESM.pdf]

Trail 1 of Fig. 1B

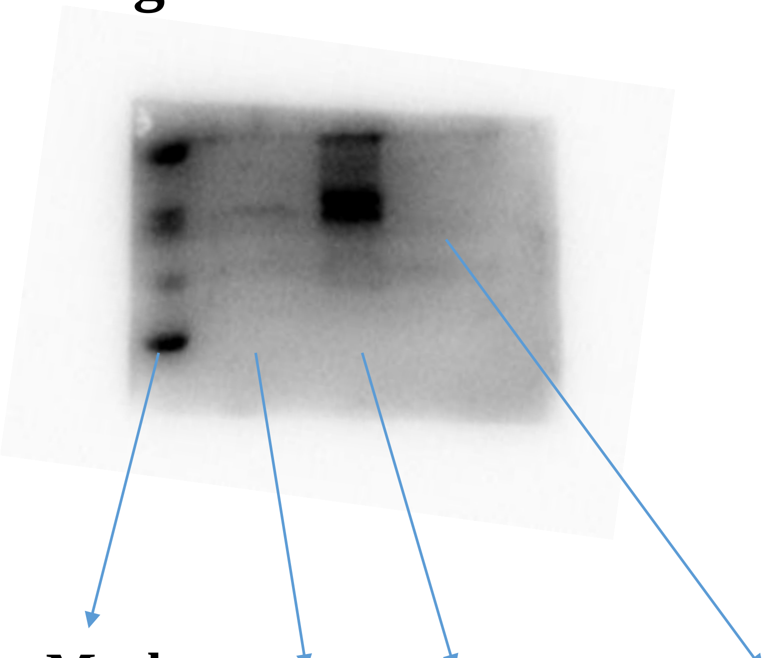

Marker Input IP: Aurora-B IgG

Trail 2 of Fig. 1B

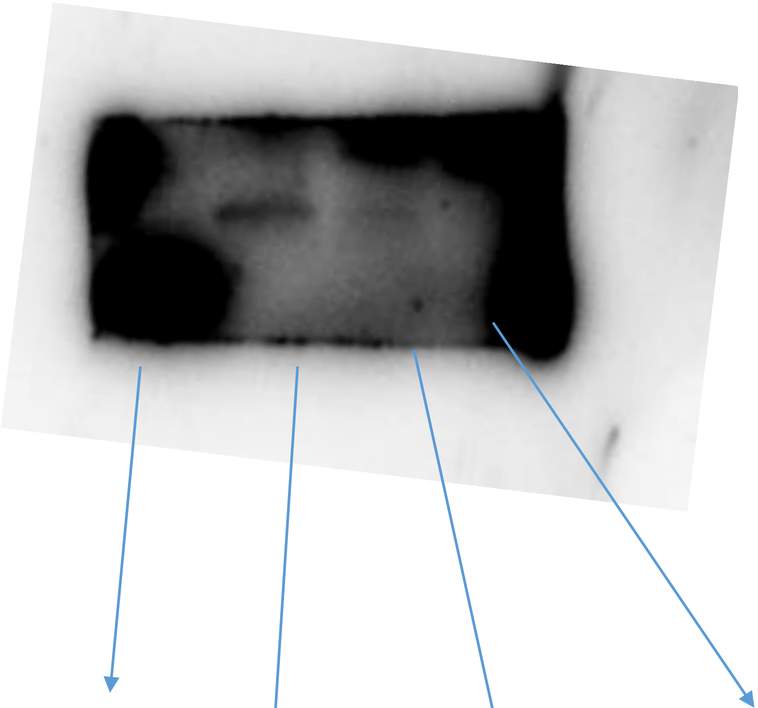

Marker Input IP: Aurora-B IgG

Trail 1 of Fig. 1C

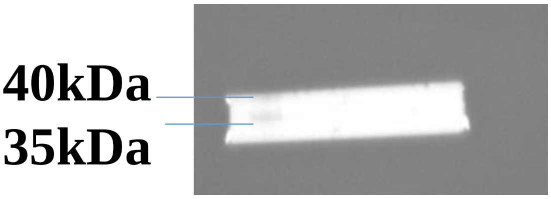

Marker-Aurora-B

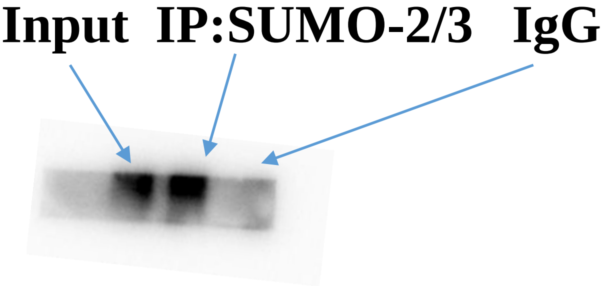

Aurora-B-30s

Trail 2 of Fig. 1C

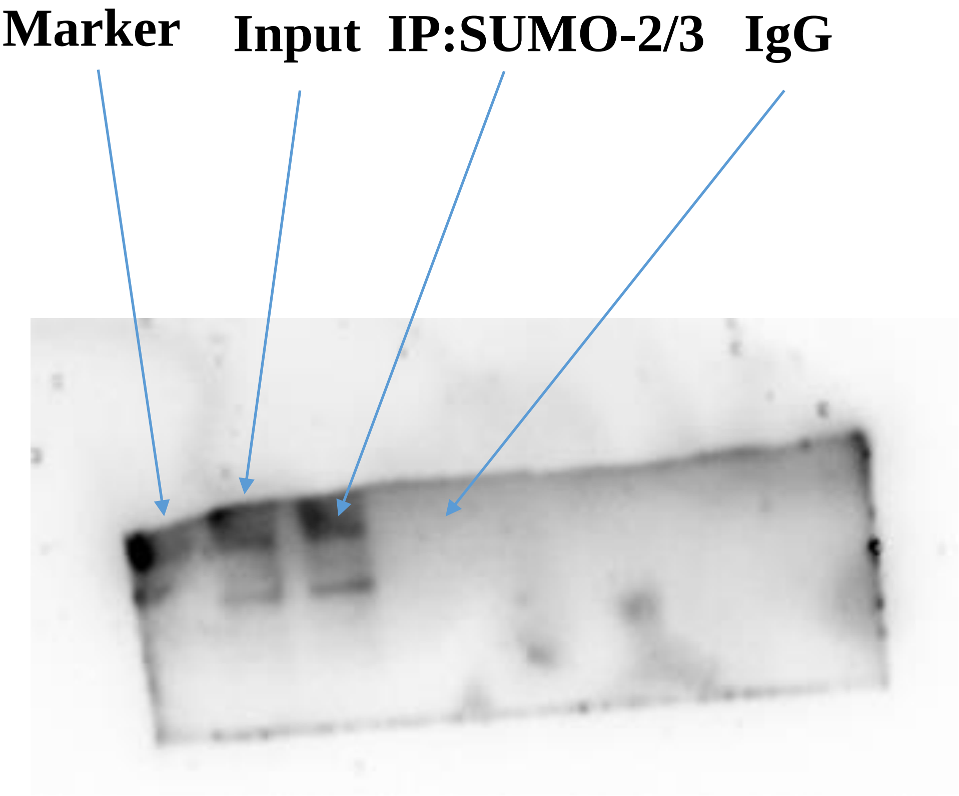

Trail 1 of Fig. 2D

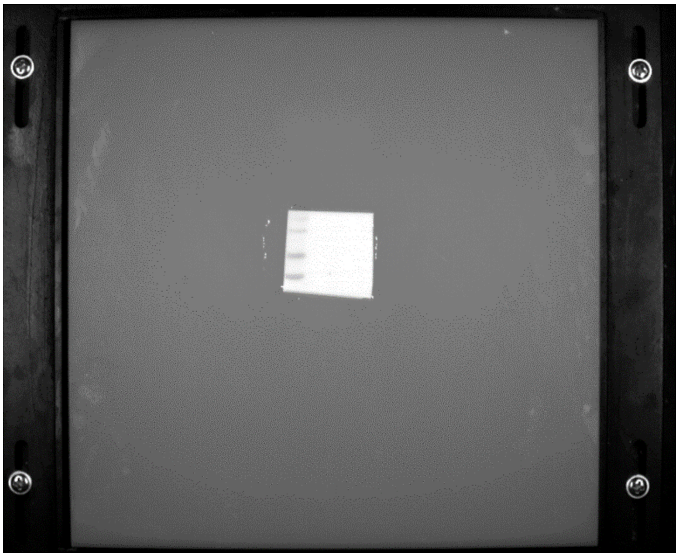

Marker-SUMO-2/3

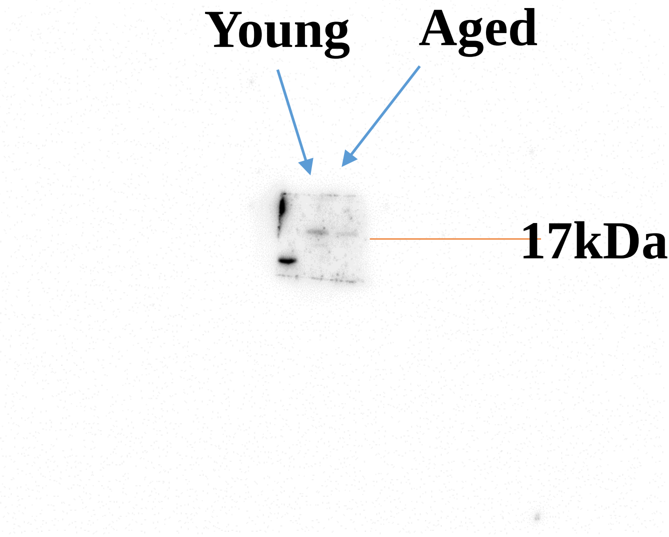

SUMO-2/3-1min

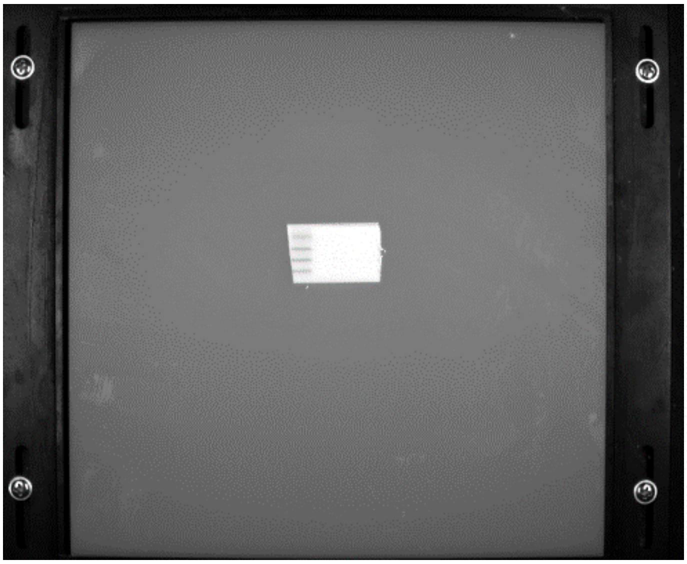

Marker-Tubulin

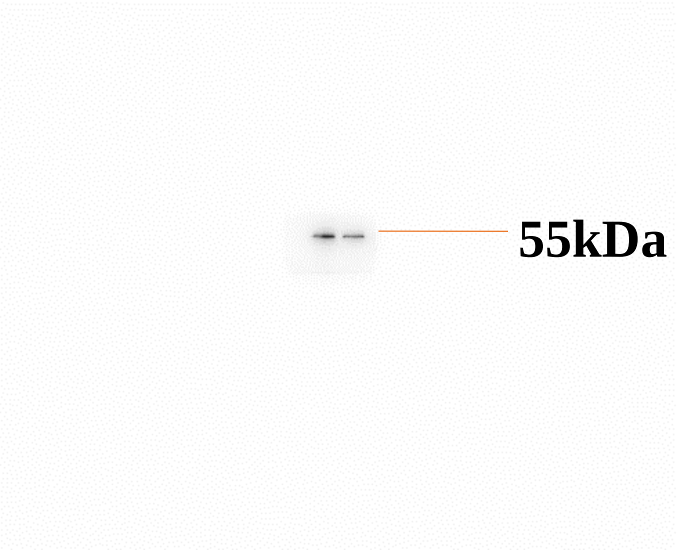

Tubulin-60S

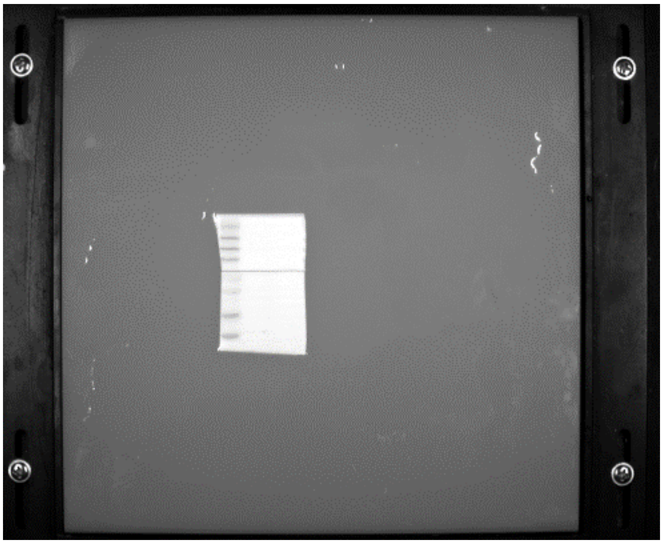

Whole-Marker

Trail 2 of Fig. 2D

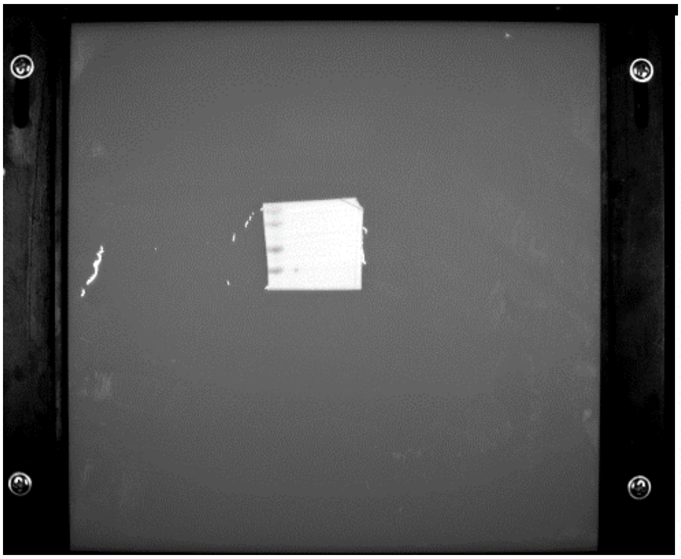

Marker-SUMO-2/3

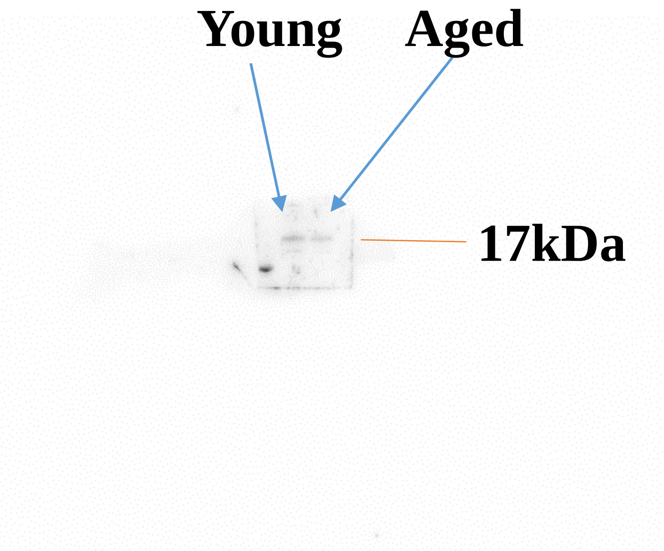

SUMO-2/3-30s

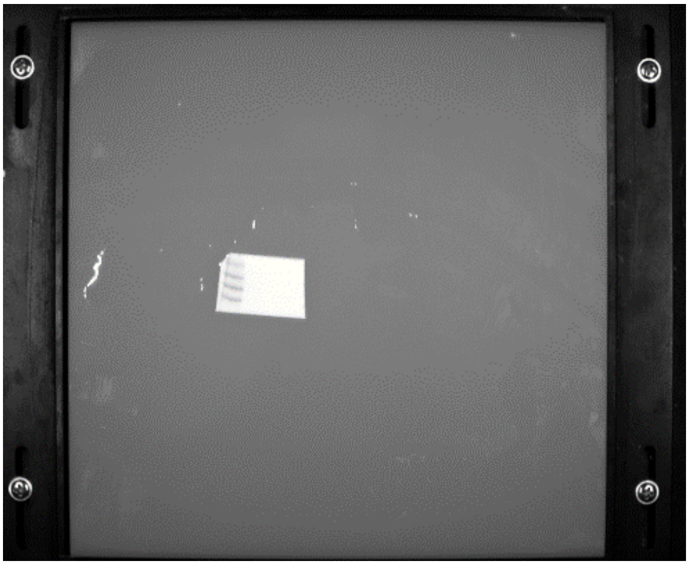

Marker-Tubulin

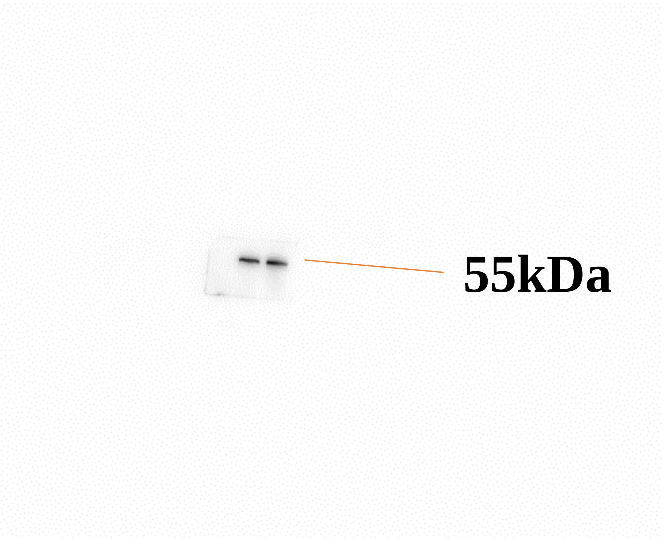

Tubulin-60s

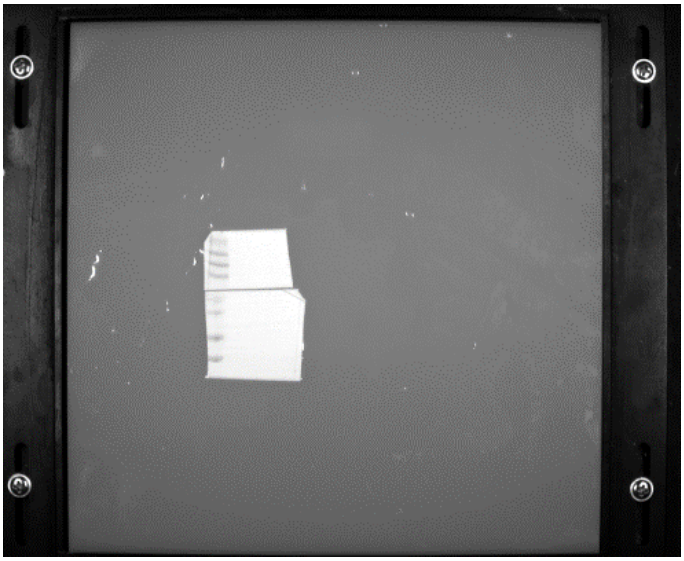

Whole-Marker

Trail 3 of Fig. 2D

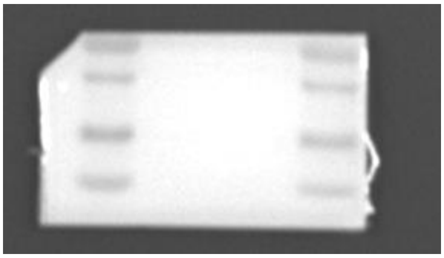

Marker-SUMO-2/3

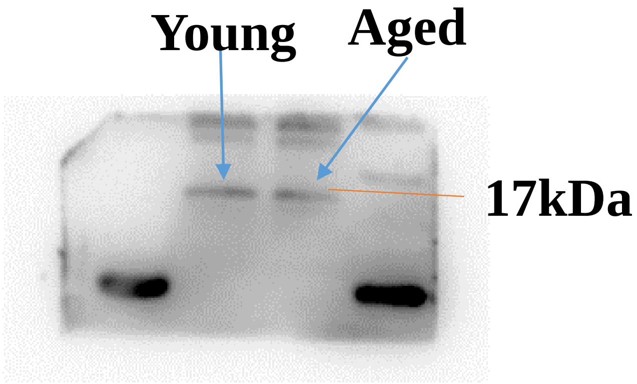

SUMO-2/3-60S

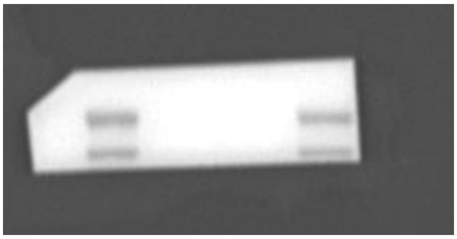

Marker-Tubulin

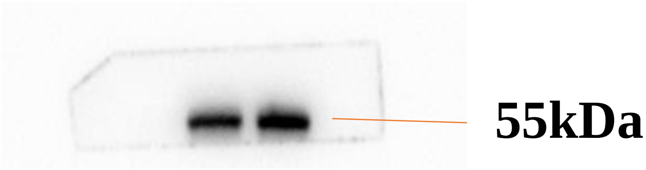

Marker-Tubulin-60S

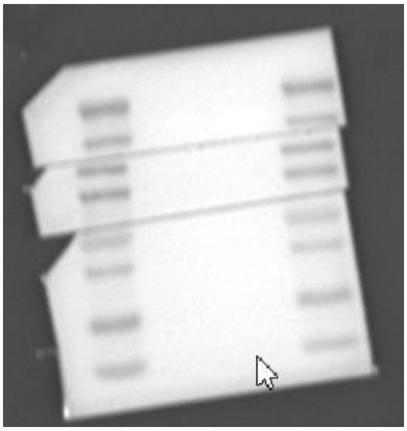

Whole-Marker
